# Dangers of the Pessary

**Published:** 1877-01

**Authors:** 


Dangers of the Pessary.—Several cases were reported. In the first, a woman 58 years old, afflicted with uterine prolapse (the organ projecting from the vulva) was immediately relieved by the application of the pessary. Soon after she fell accidentally, and the weight of the body came full upon the pessary. She suffered severe pain, which was followed in a few days by incontinence of urine. The pessary had penetrated into the bladder, and was withdrawn from the latter by Liston's forceps. Considerable loss of substance ensued, and a permanent vesico-vaginal fistula.

A second woman, 35 years of age, had some insignificant disorder six months after the birth of her ninth child, for which she consulted a charlatan. He applied a Gariel pessary, which produced violent vaginitis, with a very profuse and fetid muco-purulent discharge. The pain and the abundance and horrible odor of the discharge finally became insupportable, and as she found no relief from the advice of the would-be surgeon, she ayplied to Notta. He discovered an abscess which perforated the abdominal parietes, and was followed by an intractable fistula.

Another woman had granular endo-cervictis, with some hysterical symptoms, which improved under the author's cauterization and hydrotherapy, when by the advice of a neighbor she consulted the same charlatan. He applied the Gariel pessary, as usual, and produced a peri-uterine phlegmon, peritoneal inflammation, with abscess, which in six months destroyed her life. Notta was summoned near the conclusion of the case, and the unfortunate woman narrated to him the history of her sufferings with her own lips. Notta proceeded at once to institute judicial proceedings against the malfactor.—*L'Union Medicale.*

